# Geographic Variation in Daily Temporal Activity Patterns of a Neotropical Marsupial (*Gracilinanus agilis*)

**DOI:** 10.1371/journal.pone.0168495

**Published:** 2017-01-04

**Authors:** Emerson M. Vieira, Nícholas F. de Camargo, Paul F. Colas, Juliana F. Ribeiro, Ariovaldo P. Cruz-Neto

**Affiliations:** 1 Laboratório de Ecologia de Vertebrados, Departamento de Ecologia, Instituto de Ciências Biológicas, Universidade de Brasília, Brasília, DF, Brazil; 2 Departamento de Zoologia, Instituto de Biociências de Rio Claro, Universidade Estadual Paulista (UNESP), Rio Claro, São Paulo, SP, Brazil; Institute of Zoology, CHINA

## Abstract

The temporal activity of animals is an outcome of both biotic and abiotic factors, which may vary along the geographic range of the species. Therefore, studies conducted with a species in different localities with distinct features could elucidate how animals deal with such factors. In this study, we used live traps equipped with timing devices to investigate the temporal activity patterns of the didelphid *Gracilinanus agilis* in two dry-woodland areas of the Brazilian savanna (Cerrado). These areas were located about 660 km apart, one in Central Brazil and the other in Southeastern Brazil. We compared such patterns considering both reproductive and non-reproductive periods, and how it varies as a function of temperature on a seasonal basis. In Central Brazil, we found a constant, and temperature-independent activity during the night in both reproductive and non-reproductive periods. On the other hand, in Southeastern Brazil, we detected a constant activity during the reproductive period, but in the non-reproductive period *G*. *agilis* presented a peak of activity between two and four hours after sunset. Moreover, in this latter we found a relation between temporal activity and temperature during the autumn and spring. These differences in temporal activity between areas, observed during the non-reproductive period, might be associated with the higher seasonal variability in temperature, and lower mean temperatures in the Southeastern site in comparison to the Central one. In Southeastern Brazil, the decrease in temperature during the non-reproductive season possibly forced *G*. *agilis* to be active only at certain hours of the night. However, likely due to the reproductive activities (intensive foraging and searching for mates) this marsupial showed constant, temperature-independent activity during the night in the reproductive period at both sites.

## Introduction

The investigation of temporal activity pattern of species is an important aspect for the understanding how animals deal with several biotic (e.g., food availability, searching for mates, predation avoidance and competition) and abiotic (e.g., temperature, humidity, photoperiod length and lunar phase) factors [1]. Temporal activity patterns results from a combination of physiological constraints and ecological interaction with the environment and other organisms [2–6]. For some animals this combination may be viewed as outcomes of conflicting demands, such as the maximization of nutritional and reproductive activities, and the need to minimize costs and risks imposed by the environment such as increases in energy demand and predation [7, 8].

For small mammals, daily activity patterns are ultimately determined by the synergistic effects of predation risk [9–11], and the need of a high food intake to sustain a high metabolic rate and to cover the energy loss [12–14]. For example, studies show that distinct small mammal groups such as bats, marsupials and rodents reduce their activity in bright moon phases due to the high predation risk [15–18], in low temperatures probably avoiding energy loss through thermoregulation [3, 19, 20], and increase their activity in high-resource period peaks [20, 21].

In the Neotropics the knowledge of temporal activity of small-mammals are still limited to a coarse description, indicating only whether they are nocturnal or diurnal. Fortunately, there has been an increase of studies in the last decades that investigate the temporal activity of some species, and whether it varies according to several factors such as sex, age, competition, predation, habitat alteration, moon phases and seasonal changes [2, 4, 5, 15, 22–25]. This later should be an important aspect to investigate in relation to temporal activity of small mammals, due to the variation of abiotic factors (e.g., temperature, humidity and photoperiod length) and biotic factors (e.g., reproduction and food availability) across seasons.

Among the methods for investigating activity patterns of small mammals, there are, for example, the periodic revision of live-traps during evenly time intervals [25] and the use of radio-telemetry [26–28]. However, both methods require the presence of the researcher, and, therefore, could directly interfere on the animal activity. An alternative for these methods is the revision of the live-traps in distinct hour intervals in each night of sampling [19], but the sampling effort is reduced and several trapping events would be needed to complete all hour intervals of the day. An alternative for a method is the use of timers installed in live-traps [23, 24, 29]. This approach produces accurate time data that the animal entered in the live trap, and any bias resulted from the interference of the researcher is excluded. However, as the animals remain in the live trap until the next revision, there would be expected an exponential decrease of the number of captures along the night as suggested by Fernandez [30]. This decrease occurs mainly because the number of individuals available for trapping is reduced throughout the night [31].

**Fig 1:**
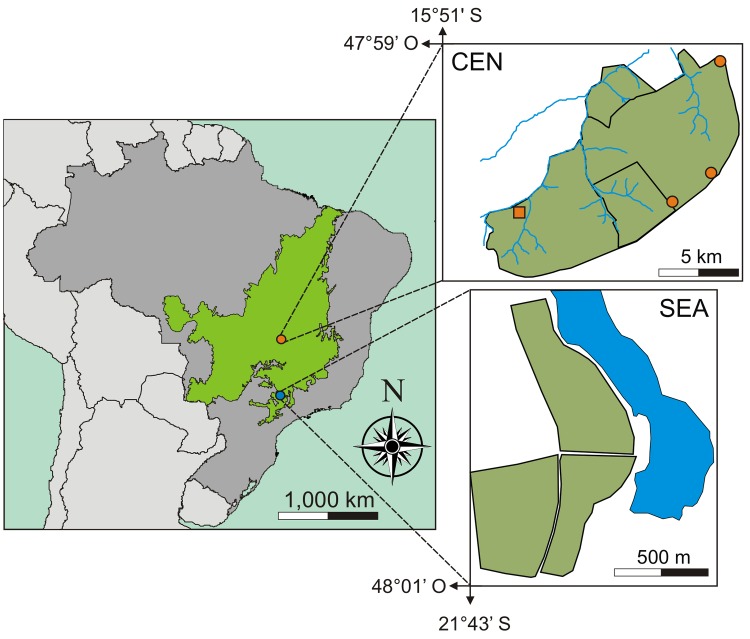
Map showing the areas of the study in the Central Brazil (CEN = orange circle) and Southeastern (SEA = blue circle) in the Cerrado (the Brazilian savanna showed as a green area in the Brazil map showed as a dark grey area). The top right inset (CEN) shows the locations of the three sampled fragments of cerradão (dry woodland) at the Botanical Garden of Brasília (orange circles in the dark green area) and one fragment at the ecological and agricultural field station of the University of Brasília (orange square in the dark green area). The lower right inset shows the sampled fragment of cerradão (dark green area) located at the municipality of Américo Brasiliense in the São Paulo State. Water bodies are shown in blue in the CEN and SEA insets.

In this study we investigated the temporal activity of the gracile mouse opossum *Gracilinanus agilis* (Burmeister, 1854) in dry woodland forest (cerradão), a xeromorphic forest-like physiognomy of the Brazilian savanna (Cerrado). Additionally, we propose a new method for correcting capture-related potential bias on evaluation of small-mammal daily activity based on live trapping. Assuming the potential role of biotic and abiotic factors in the temporal activity patterns of this didelphid, our study was conducted in two sites with distinct climatic features that were located in the border of the Cerrado (Southeastern Brazil) and in the core area of this Biome (Central Brazil) (Fig 1). Lower temperatures, especially in the winter, and a more distributed precipitation along the year are expected in in the Cerrado of Southeastern Brazil (SEA) in comparison to the Cerrado of Central Brazil (CEN), since both areas are located about 660 km apart and in distinct climatic regions [32] according to Köppen’s classification [33] (SEA = Maritime temperate climates [Cwb]; CEN = winter dry season tropical savanna [Aw]). Taking these differences between sites into consideration, our objectives were: 1) to compare the temporal activity patterns of *G*. *agilis* between the reproductive and non-reproductive seasons; 2) to evaluate whether there is a relation between temperature and activity level of this marsupial; and 3) to evaluate whether the patterns are similar in the two distinct regions of the Cerrado. Our expectations were: 1) *G*. *agilis* changes its temporal activity patterns across reproductive season. More specifically, due to necessity of searching for mates and the need of high-energy intake for reproduction [34], we expected a more constant activity during the night in the reproductive season. On the other hand, in the non-reproductive season we expected *G*. *agilis* to be more active in optimal periods (e.g., warmer hours, periods with less predation risk, and periods with high resource availability); 2) we anticipate a relationship between temperature and intensity of activity by *G*. *agilis* along the year, since this marsupial would be more active in warm periods. Being more active such periods could be advantageous for avoiding a potential energy loss through thermoregulation [35] and also could increase foraging success, since arthropods, an important food source for *G*. *agilis* [36], are potentially more active in warm periods [37]; 3) Moreover, we expected differences in daily activity patterns between sites of woodland forests. Due to the differences in climatic factors, we would expect temporal activity to be less affected by temperature in the forests of Central Brazil, where climatic seasonal differences are less marked than in the Southeastern Brazil. This is expected because the didelphid *G*. *agilis* tend to enter in torpor in temperatures below 20°C [38]. Therefore a total or partial reduction of *G*. *agilis* activity in the lower temperatures of the Cerrado of Southeastern Brazil should occur.

## Methods

### Ethics Statement

The Institutional Animal Care and Use Committee of the Universidade de Brasília (CEUA—UnB) approved this research (approval No. 44743/2009). All the methods used in this study related to capture and handling of the animals comply with the guidelines recommended by the American Society of Mammalogists [39] and with the requirements of The Brazilian Institute for the Environment and Natural Resources (IBAMA) (Permit No. 15424–1, IBAMA Registration No. 15778628). Permissions for field data collections were given by Segundo Ungari Neto (Clube Náutico Araquara), by the director of the ecological and agricultural field station of the University of Brasília (FAL), José Mauro da Silva Diogo, and by the technical and scientific superintendent of the Botanical Garden of Brasília, Vânia de Araújo Soares. The field studies did not evolve endangered or protected species.

### Studied species

*Gracilinanus agilis* is a small (body mass = 20–30 g), nocturnal and scansorial didelphid marsupial that occurs from the border of Panama with Colombia to the Northeast, Midwest and Southeast of Brazil [40]. It is generally common in gallery forests (evergreen vegetation that surrounds streams) and cerradão areas in the Brazilian Cerrado [41, 42]. This marsupial has a seasonal pattern of reproduction, with the presence of pregnant and lactating females from September to March [43].

Previous studies conducted in Southeastern Brazil, in the same Cerradão area that we sampled in the presented study [43, 44], refer to this gracile mouse opossum as *Gracilinanus microtarsus*. However, physiological studies conducted with the same population [38, 45], specimen descriptions, distribution and habitat use lead us to accept the suggestion that the studied species is *G*. *agilis* (Leonora Costa, Universidade Federal do Espírito Santo, pers. comm.; see also reference [46]). Indeed the available specimens from this region were reexamined and on the basis of morphological traits recently published in references [47–49] and identified as *G*. *agilis* (PFC, unpub. data).

### Studied area

#### General characterization

The study took place in sites of cerradão in both Southeastern (SEA) and Central (CEN) Brazil. The cerradão is comprised by arboreal species of gallery forests and also by tree species from cerrado *sensu stricto* (typical savanna habitat), presenting a tree layer that varies from 8–15 m, and the tree cover ranges from 50–90% [50]. In the SEA, we collected field data in a single fragment of 307 ha (21°43' S, 48°01' W) at the Clube Náutico Araraquara, a private area located in the municipality of Américo Brasiliense in the São Paulo State. In the CEN, we conducted the study in three sites at the Botanical Garden of Brasília (15°52' S, 47°50' W) and one site at the ecological and agricultural field station of the University of Brasília (15°58′ S, 47°59′ W), located near the city of Brasília in the Federal District of Brazil. These two locations are part of a legally protected area that covers about 8,800 ha of typical core area of Cerrado vegetation. The four sampled sites were relatively close to each other (distance between them ranged from 3.0 to 15.5 km; see reference [51] for details on these sites) and subject to similar climatic conditions.

#### Climate characterization

**Fig 2:**
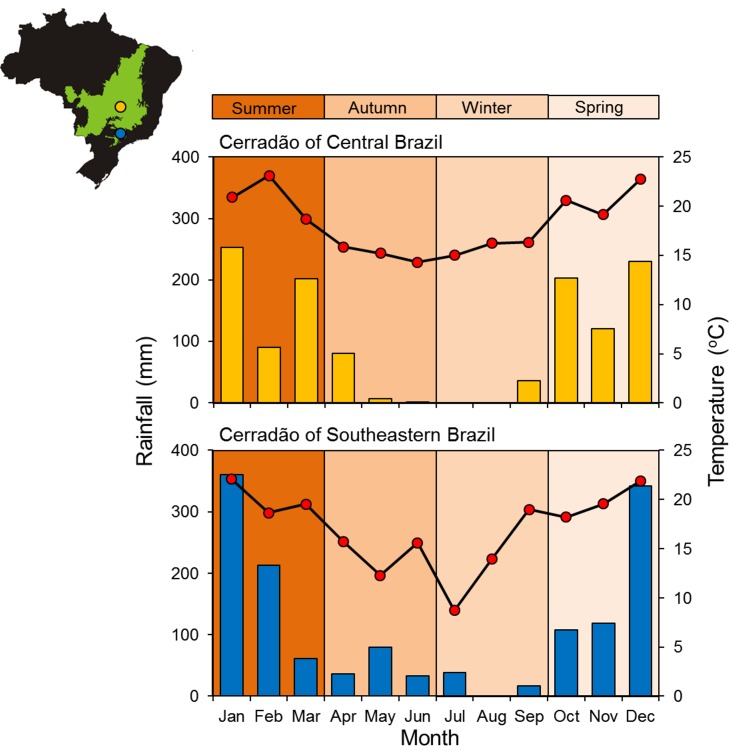
Monthly total rainfall (orange and blue bars) and mean temperature (red circles) of the studied areas of dry woodland (cerradão) from the Central Brazil (CEN) and Southeastern (SEA). Seasons of the year on the top of the graphic are represented according to the corresponding months indicated in the x-axis. The Brazil map shows the localities of the areas of the study in the CEN (orange circle) and SEA (blue circle) in the Cerrado (green area).

The study area in Central Brazil (CEN) is located on the great plateau of Central Brazil, in the core area of the Cerrado distribution. This biome presents two well-marked seasons: the cool-dry and the warm-wet seasons [52]. This later falls between October and April, in which 90% of the annual precipitation of 1100–1600 mm occurs [53]. On the other hand, the SEA is part of disjunct areas of Cerrado located at higher latitudes in the border the biome (Fig 1). For that reason, in this Southeastern area the two seasons of the Cerrado are less pronounced and influenced by a more temperate climate in comparison to the core area of the biome. Fig 2 shows this trend according to mean temperature and total rainfall in the months of the study period (see also *Abiotic factors* in the *Data collection* section).

### Data Collection

#### Animal captures

Animal captures took place from January to December 2004 in the SEA. We conducted monthly field sessions of five consecutive nights, using 100 Sherman live-traps in a grid composed by 100 capture stations (10 x 10), spaced at 10-m intervals (totaling 6,000 trap-nights). Among these traps, 63 were equipped with digital timers that indicated the time of capture to the nearest minute. Each live-trap was placed at 2-m high on three branches and baited with cod liver oil.

We conducted capture sessions in the CEN from September 2009 to August 2010 using Sherman live traps placed in three grids of 144 (12 x 12) and one of 108 (9 x 12) capture stations spaced at 15-m intervals. We sampled each grid six times (every two months) using 80 live traps placed on the ground and 80 in the understory (1.5–2.5 m) in randomly selected capture stations. Among these traps, 60 were equipped with digital timers as in the SEA traps. Each capture session lasted six consecutive nights (totaling 23,040 trap-nights) and the Sherman live traps were baited with a mixture of peanut butter, vanilla essence, maize flour, cod liver oil, and banana.

We registered date and time of capture and reproductive condition of females, which was inferred mainly from the swelling of the nipples [54]. We did not evaluate the reproductive condition of males because of inaccurate evaluation of such condition based on external characteristics [55]. Each individual received a numbered plastic leg-band or an ear-tag (1005–1, National Band and Tag, Newport, Kentucky) and was released at the point of capture after data collection.

#### Abiotic factors

During each capture session in both SEA and CEN we used a data logger model HOBO^®^ (Onset Computer Corporation, Bourne, Massachusetts, USA; -10°C to 50°C, to the nearest 0.1°C) attached on the vegetation branches to obtain the daily temperature every 15 minutes. For the SEA, we obtained the monthly precipitation data from the pluviometric database from the Integrated Water Resources Management of São Paulo [56], for the Araraquara municipality (approximately 20 km from the study area). For the CEN, we obtained the pluviometric data assessing the Meteorological Database for Education and Research (Banco de Dados Meteorológicos para Ensino e Pesquisa) of the National Institute of Meteorology (Instituto Nacional de Meteorologia) [57]. These data were obtained from the meteorological station of Roncador, located between 2.5 and 9.0 km from the study sites. For statistical purposes (see *Statistical analysis* for more details), we also obtained the time data of the sunrise and sunset to the nearest minute from each locality for the period of the study. These data are available in the Interactive Yearbook of the National Observatory (Observatório Nacional, 2016) [58].

### Data analysis

Considering that *G*. *agilis* is exclusively nocturnal and due to seasonal differences in the photoperiod length, we pooled the monthly captures of *G*. *agilis* considering 2-h intervals after sunset (first half of the night) and before sunrise (second half of the night), resulting in 6 time intervals. However, due to differences in the photoperiod length, the interval correspondent to 6 hours before sunrise lasted from 31 min to 3 h and 11 min in the SEA, while in the CEN lasted from 1h20min to 3h03min. To reduce the effect of this variation, we corrected the number of captures of this interval by the length of this category, thus estimating the number of captures on a 2-h basis using the cross-multiplication method. For the data obtained in the distinct sites of CEN, we pooled all captures in 2-h intervals regardless the site.

For correcting the potential bias involving the exponential decrease of the number of captures along the night because trapped animals are not available for subsequent recapture [30, 31], we propose a method of correction to obtain an estimated number of captures of *G*. *agilis* considering the 2-h intervals, calculated as:

$$C_{\mathrm{corr}}=[C/(E\;\text{-}\;\Sigma C_{\mathrm{ant}})]\;x\;E$$ (1)

where C_corr_ is the corrected number of captures, C is the number of captures in a given 2-h interval, E is the estimated population size in the trapping session, and ΣC_ant_ is the sum of captures of all previous 2-h intervals in the same night. Therefore, this method takes into consideration the total size of population, the number of previously captured animals in the same night and the potential number of individuals active in each 2-h interval. For the distinct localities of Central Brazil, we performed this correction before pooling the data in 2-hour intervals, since each site was occupied by independent populations. For estimating the population size (E) of *G*. *agilis* from both study areas, we used monthly capture-mark-recapture data for calculating the Minimum Number Known Alive (MNKA; [59]).

We compared temporal activity patterns between reproductive and non- reproductive periods with a Kolmogorov-Smirnov two-sample test using the software BioEstat 5.0 [60]. For that, we pooled the corrected number of captures of each 2-h interval of all individuals from the months in which females indicated to be reproductively active. For this analysis, we did not consider the last month in which females presented external characteristics of reproduction. Since few individuals present external evidence of reproductive condition in this period, it is likely that potentially distinct temporal activity patterns of reproductive individuals would be biased by the non-reproductive ones. On the other hand, we pooled the data from the month just before reproductive females were present in the populations with the reproductive months. Because of the rapid change of the reproductive condition in females (100% of the individuals being pregnant or lactating from one month to another as seen in reference [43]), we assumed that males would already be performing their reproductive activities in this preceding month (i.e., dispersing and intensively searching for mates).

Besides performing the analysis comparing reproductive and non-reproductive periods within each area (SEA and CEN) we also contrasted the activity patterns of *G*. *agilis* between the same periods of each area, also using K-S tests. Because of the lack of differences in activity patterns between males (number of registers [N] = 76) and females (N = 80) found in the SEA (Kolmogorov-Smirnov test: D = 0.11, P = 0.641), and the insufficient data for similar comparison in the CEN (15 males and 63 females), we lumped both sexes together for the analyses. For all the analyses, we used only data from the first capture of each individual during each capture session.

To evaluate the influence of temperature in the temporal activity of *G*. *agilis*, we performed a logistic regression between presence and absence of captures during each 1-h interval from 1800 to 0600 hrs (dependent variable) along the capture sessions of each year season and the average of four measures of temperature (°C) during each of those 1-h intervals (independent variable; as in reference [23]). We used a logistic regression because the number of captures per 1-hour interval was represented mainly by 0 (no captures) or 1 (88% of the 1-h intervals). We accounted for intervals with multiple captures by repeating that occurrence with the corresponding temperature as much as the number of captures of the interval. We performed this analysis grouping the months corresponding to the four seasons of the year (see Fig 2). Even though the CEN presents a typical Cerrado pattern of two pronounced seasons during the year (i.e., warm-wet and cool-dry) of the Cerrado, we also grouped the data according to the classic four seasons of the year for direct comparison with the SEA area, which presents characteristics of a more temperate climate (i.e., more marked differences among the four seasons) in comparison to the CEN.

We graphically compared the average temperature of each season using the average temperature of 1-hour intervals of each month for showing the distinction in temperature patterns between the SEA and CEN. We also contrasted the temperatures considering the reproductive and non-reproductive periods of *G*. *agilis*.

## Results

During the study we obtained 156 records (102 from the non-reproductive period and 54 from the reproductive period) of capture times of *G*. *agilis* in the SEA. In the CEN, we obtained 78 records (49 from the non-reproductive period and 29 from the reproductive period). Based on our data regarding external characteristics indicative of female reproductive condition, in both areas *G*. *agilis* was reproductive from September to January.

**Fig 3:**
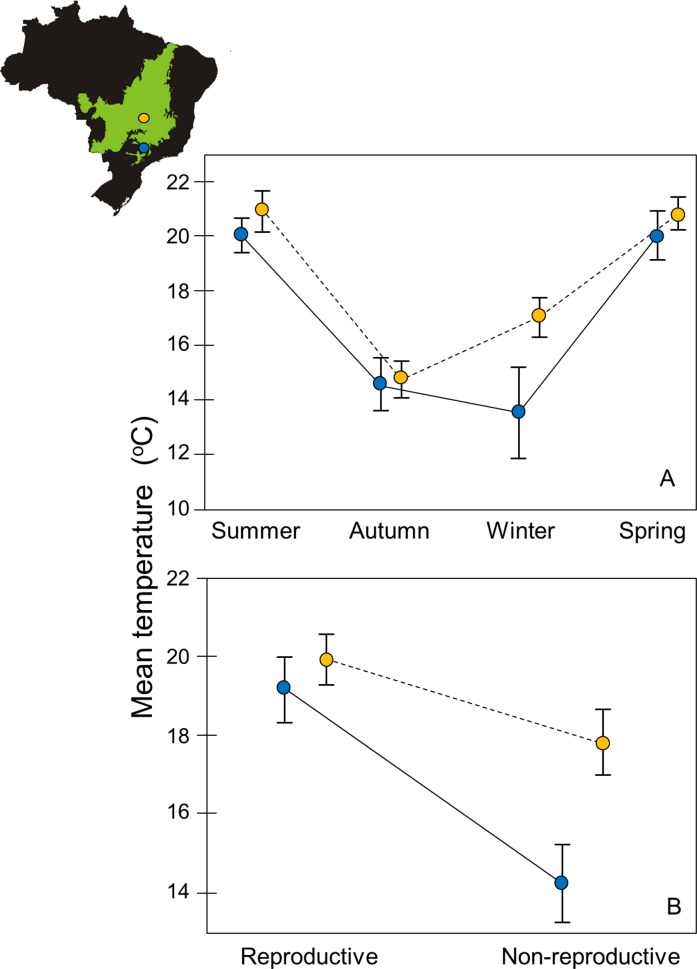
**Comparison of the night mean temperature of 1-h intervals (from 1800 to 0600 hrs) between the cerradão (dry woodland forest) of Central Brazil (orange circles) and Southeastern Brazil (blue circles) considering the four seasons of the year (A) and the reproductive and non-reproductive periods of *Gracilinanus agilis* (B).** Vertical bars show the standard deviation. The Brazil map shows the localities of the areas of the study in the CEN (orange circle) and SEA (blue circle) in the Cerrado (green area).

Our comparisons of temperature across seasons between the both regions indicated that with the exception of the winter, in which the Southeastern area was cooler than the area located in Central Brazil, the other seasons do not differ between areas. Additionally, there was a clear difference between both areas during the non-reproductive season, when the temperature was lower in the SEA in comparison to the CEN. This difference, however, did not hold for the reproductive period. The reproduction of *G*. *agilis* occurs in warmer and similar temperatures when the two areas are compared (Fig 3).

**Fig 4:**
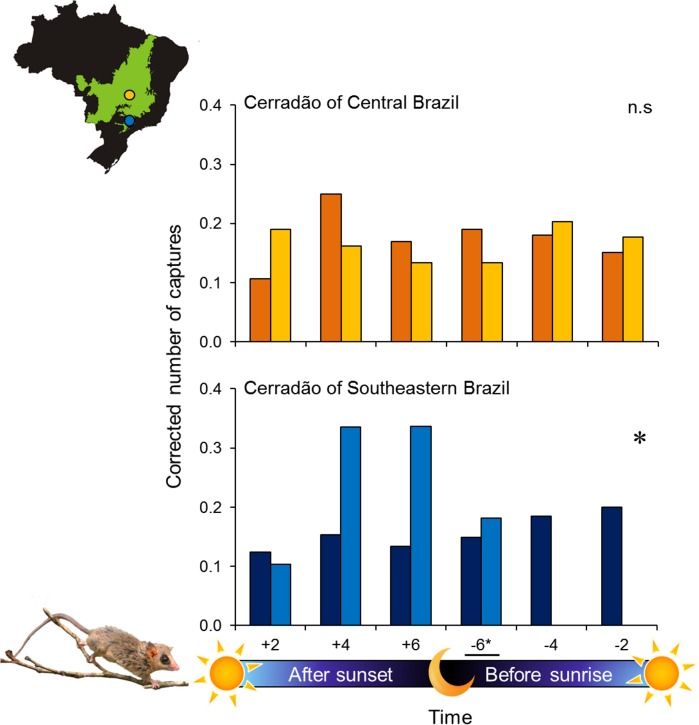
Comparison of temporal activity pattern of *G*. *agilis* according to reproductive (R) and non-reproductive (NR) periods of dry woodland (cerradão) areas from the Central Brazil (R = dark orange; NR = light orange) and Southeastern Brazil (R = dark blue; NR = light blue). The asterisk in the low chart indicates significantly distinct temporal activity patterns between reproductive and non-reproductive periods (P<0.05; Kolmogorov-Smirnov two sample test). ‘n.s’ in the top chart indicates no significant differences in the temporal activity patterns between reproductive and non-reproductive periods. The line under 6 hours before sunset indicates the 2-h interval in which the number of captures was adjusted according to the photoperiod length (see *Data analysis* in *Methods* for more details). The Brazil map shows the localities of the areas of the study in the CEN (orange circle) and SEA (blue circle) in the Cerrado (green area).

Our analysis comparing temporal activity of *G*. *agilis* between the reproductive and the non-reproductive periods showed differences in the SEA (D = 0.472; P = 0.030) but not in the CEN (D = 0.160; P = 0.490), with a clear activity peak in the first part of the night during the non-reproductive period only in the SEA (Fig 4). Comparing the reproductive period between SEA and CEN we found no differences in temporal activity of *G*. *agilis* (D = 0.214; P = 0.466). On the other hand, in the non-reproductive period, the early-night peak observed in the SEA also caused differences in temporal activity in comparison to a more regular nocturnal activity in CEN (D = 0.381; P = 0.018).

We detected a positive and significant relation between temperature and activity level of *G*. *agilis* in the spring and in the autumn in the SEA. On the other hand, in the CEN we did not find any relationship between temperature and activity in any of the seasons of the year (Table 1).

**Table 1:** Results of the relation (logistic regression) between air temperature and captures of *Gracilinanus agilis* during one-hour periods for each season in dry-woodland (cerradão) areas of the Southeastern and Central Brazil.

Season	NP	Z-value	Odds ratio	P-value
Southeastern Brazil				
Summer	64	-0.922	0.865	0.357
Autumn	61	3.135	1.725	**0.002**
Winter	48	-0.563	0.969	0.573
Spring	58	2.950	1.592	**0.003**
Central Brazil				
Summer	48	-0.112	0.985	0.911
Autumn	29	0.917	1.226	0.358
Winter	35	1.755	1.493	0.071
Spring	42	1.516	1.516	0.110

NP = number of periods for each season on which the analysis were based. Bold values in P-value column indicate significant relationship between air temperature and captures (P < 0.05).

## Discussion

In this study we investigated the temporal activity patterns of *G*. *agilis* in relation to reproduction and temperature in two dry-woodland areas of the Brazilian savanna (Cerrado) located about 660 km apart. Despite the fact that our study was carried out with the same marsupial species and in in the same forest formation of the Cerrado, we detected distinct trends in temporal activity patterns. The daily activity of *G*. *agili*s differed between the reproductive and the non-reproductive periods only in the region with lower annual temperatures, in Southeastern Brazil (SEA). Moreover ambient temperature influenced on the activity level of this marsupial only in the SEA. The observed results reinforce the relevance of abiotic factors in determining temporal activity of small mammals.

For conducting this study, we relied on digital timers attached to traps to assess the exact capture time, as carried out in several other field studies on small-mammal temporal activity patterns [23, 24, 29]. This method is, however, subject to criticisms mainly because the captured animals remain inside the traps and not only these traps are not available to document activity at latter periods but also the overall number of trappable animals is reduced through the night. Different from previously suggested approaches [31], the correction formula that we used applied different weights to captures that occurred in different periods along the same night and also considered the total population size of the animals in the trapping grids. We believe that our correction eliminated or greatly reduced these potential biases but they would not be relevant in the present study because of two reasons. First, the main spurious effect of the trapping protocol on mammal activity would be and activity peak in the beginning of the activity period and a gradual decrease along the night, which we did not observe in our results. Second, as we compared activity patterns across seasons and between areas using similar trapping protocols the influence of any systematic bias in detecting activity on our comparisons is negligible.

Our results showed that while in the SEA *G*. *agilis* presents a fairly constant activity during the night in the reproductive period, this marsupial presents a marked peak of activity in the non-reproductive one. On the other hand, in the area located in Central Brazil (CEN), *G*. *agilis* presents a fairly constant nocturnal activity during both non-reproductive and reproductive periods. Possibly the observed pattern of constant activity during the reproductive period in both studied areas is a result of the increasing food intake by both sexes to meet their reproductive needs. This is supported by the general pattern of reproductive costs indicated for small mammals, in which males face high energetic costs searching for females, while females face a high energetic cost during gestation and lactation [34]. Therefore, individuals should increase their foraging activities during this period. Extensions of activity period for reproductive individuals are not uncommon, and were observed for example, in the Australian dasyurid *Antechinus stuartii* Macleay, 1841 [61], in the European ground squirrel *Spermophilus citellus* (Linnaeus, 1766) [62], and in the water opossum from the Brazilian Atlantic Forest *Chironectes minimus* (Zimmermann, 1780) [63].

In the SEA, the activity of *G*. *agilis* during the non-reproductive period was concentrated mainly during the intervals of four and six hours after the sunset. Since individuals were not dispersing or facing high energetic demands for reproduction, these intervals probably represent optimal hours of the night to be active searching for food. Among relevant factors that can characterize optimal hours for small mammals are, for example, periods in which predators are less active [2, 64] or else periods with suitable temperatures (i.e., non-extreme thermal conditions; [65, 66]). The activity of predators such as owls [67] or carnivorous bats [68] seems not to be the main factor influencing the temporal activity of *G*. *agilis* in this study, as the patterns found in the non-reproductive season differed between SEA and CEN. Therefore, the temperature possibly plays an important role on the activity pattern of *G*. *agilis*. This idea is reinforced by our between-area temperature comparisons, which show lower temperatures in the SEA in comparison to the CEN during the non-reproductive period. While in the SEA the average night temperature during the non-reproductive period is around 14°C, in the CEN is around 18°C. Since *G*. *agilis* enters in torpor in temperatures below 20°C [38], it is expected that this marsupial reduces partially or totally its activity in periods with low temperatures.

Additionally, our results showed a positive relationship between temperature and activity level of *G*. *agilis* only in Southeastern Brazil. This pattern also reinforces the importance of temperature on the activity of this marsupial, and such behavior probably minimizes energy loss by thermoregulation [38]. Moreover, in warm periods *G*. *agilis* also could benefit from the greater activity of arthropods [37], an important food source for this marsupial [36]. On the other hand, the mild temperatures in Central Brazil might reduce physiological constraints and allow this marsupial to be constantly active during the night even during winter, in the non-reproductive period.

A regular activity pattern in the cooler non-reproductive period in Central Brazil could be advantageous, for example, in searching and using high-quality resources [69]. A similar pattern, although related to seasons, was observed, for example, for the Mexican fox squirrel *Sciurus nayaritensis chiricahuae* Goldman, 1933. This species shifts from an expanded and bimodal activity in the morning and in the afternoon during summer to a reduced and unimodal activity in the middle of the day in winter [70]. Additionally, the European badger *Meles meles* (Linnaeus, 1758) show biogeographical differences in the annual cycle of activity according to climatic features. This animal can be more active year round in regions with warm climates, whereas in regions with severe climatic conditions badgers are inactive during the winter, active mainly at dusk and down during the autumn, and constantly active during the summer [71].

The role of temperature on the temporal activity of small mammals has been pointed out in other studies [19, 23, 24] and is possibly related to other factors such as the size of the animal and the range of temperature variation [12]. For example, the activity of the large didelphid *Didelphis aurita* (Wied-Neuwied, 1826) (average mass = 1.23 kg; [72]) seems not to be limited by low temperatures (mean minimum temperature = 4.5°C) in the Brazilian Atlantic forest [22]. On the other hand, the congeneric *D*. *virginiana* Kerr, 1792 (average mass = 2.02 kg; [73]) is less active during low and severe temperatures (mean minimum temperature of = -8.8°C) in Ithaca, New York [74]. However, *G*. *agilis* is about 40 times smaller than the *D*. *aurita* and probably this small marsupial is potentially more sensitive even to low temperature variations and climatic differences between localities, as seen in the studied areas.

Although we found a relationship between temperature and activity level by *G*. *agilis* in the SEA, this pattern occurred only during the autumn and the spring. One possible explanation for such pattern is that autumn and spring are seasons with intermediate temperatures in comparison to summer and winter, and, therefore, the relationship between temperature and intensity of activity arises. On the other hand, the lack of relationship in the summer could be attributed to the high abundance of young individuals in the population during this season [43]. Distinct temporal activity between adult and young individuals of *D*. *viriginiana* [75] and *D*. *aurita* [22] was verified in other studies, and, therefore, it is possible that the influence of the temperature was diluted within the population during our analysis. Moreover, the lack of relationship between temperature and temporal activity in the winter could be attributed to the fact that in the end of this season, *G*. *agilis* is starting its reproductive activities [43]. Possibly during the reproductive period this marsupial tends to be more intensively and constantly active along the night, thus resulting in the lack of relationship between temperature and temporal activity. According to reference [43], in the last month of the winter (September) and for three consecutive years, 100% of the females from the same population of our study were lactating or pregnant. Therefore, it is also reasonable to assume that reproductive activity and resulting changes in daily activity patterns occurred earlier in this season. In addition to the temperature, it is also possible that precipitation plays a role in the temporal activity of *G*. *agilis* (e.g., [76, 77]). In our study, however, we were not able to evaluate detailed responses of this marsupial to rainfall since we could not get hourly data for precipitation as we did for temperature.

## Conclusions

Our study revealed that the patterns of daily temporal activity of *G*. *agilis* in dry woodland areas show distinct trends in geographically distinct regions of the Brazilian Cerrado. In Central Brazil (CEN), *G*. *agilis* presented a fairly constant activity along the night in both reproductive and non-reproductive periods. However, in an area located in the Southeastern Brazil (SEA), in the border of the Cerrado distribution this marsupial showed differences in temporal activity patterns along the year. In this latter area this species was constantly active in the reproductive period but not in the non-reproductive one, when a peak of activity between two and four hours after the sunset occurred. We suggest that the observed differences in temporal activity between areas are related to more pronounced climatic differences in the SEA across seasons, in addition to overall lower temperatures in comparison to the CEN. Therefore, in this latter, during the reproductive period *G*. *agilis* is probably more active searching for mates and foraging than in the non-reproductive period. On the other hand, during this latter period this marsupial tends to increase its activity in optimal hours, probably avoiding potentially costs related to thermoregulation and/or synchronizing its activity with that of invertebrate prey (i.e., warmer periods along the night). This idea is reinforced by the positive relationships between temperature and activity intensity observed only in the dry woodland area of the SEA. Chronoecological studies conducted in distinct localities and with distinct environmental features can be an important approach for enlightening how small mammals deal with constraints of conflict demands, which can be related to both biotic and abiotic factors.

## Supporting Information

S1 Dataset Temporal activity data of the opossum *Gracilinanus agilis*. (XLSX)
